# Node Deployment Algorithm Based on Viscous Fluid Model for Wireless Sensor Networks

**DOI:** 10.1155/2014/350789

**Published:** 2014-07-14

**Authors:** Jiguang Chen, Huanyan Qian

**Affiliations:** School of Computer Science and Engineering, Nanjing University of Science and Technology, Nanjing 21094, China

## Abstract

With the scale expands, traditional deployment algorithms are becoming increasingly complicated than before, which are no longer fit for sensor networks. In order to reduce the complexity, we propose a node deployment algorithm based on viscous fluid model. In wireless sensor networks, sensor nodes are abstracted as fluid particles. Similar to the diffusion and self-propagation behavior of fluid particles, sensor nodes realize deployment in unknown region following the motion rules of fluid. Simulation results show that our algorithm archives good coverage rate and homogeneity in large-scale sensor networks.

## 1. Introduction

As one of the basic problems in wireless sensor networks, deployment algorithm has attracted scholars' wide attention, while a series of algorithms have been put forward in allusion to different deployment demand. In this paper, with regard to the problem of large-scale wireless sensor network deployment, a scheme based on viscous fluid model is proposed.

A large number of researches have been done to solve various issues related to the deployment problem. There are practically three categories of methods: methods based on geometrical model, methods based on virtual potential field, and methods based on biological intelligence. Since fluid field is one kind of virtual potential field, the algorithm proposed in this paper belongs to methods based on virtual potential field.

The most classical method based on virtual potential field is proposed by Howard et al. [1]. They provided a solution to the problem by deploying a mobile sensor network in unknown dynamic environments. Moreover, they described a potential-field-based approach for deployment, in which nodes were treated as virtual particles, subject to virtual forces. These forces repel nodes from each other and from obstacles, ensuring that, from an initial compact configuration, nodes will spread out to maximize the coverage area of the network. In addition to these repulsive forces, nodes are also subject to a viscous friction force. This force is used to ensure that the network will eventually reach the state of static equilibrium; that is, all nodes will ultimately come to a complete stop. Similarly, the virtual force algorithm of [2] and the virtual spring force algorithm of [3] use both repulsive and attractive force components to maximize coverage and uniformity of a given number of sensors.

As we know, electrostatic field also is a kind of virtual potential field. In [4], Toumpis and Tassiulas imagined a scenario: the spatial distribution of sources and sinks is fixed, but we are free to place wireless nodes as we like. Then they raised a question: how to calculate the minimum number of nodes so as to support the traffic, as well as the associated placement of nodes that achieves this minimum. Apparently, it is plausible to solve this problem with regular approach. Yet, this may lead to many troubles and takes more time. By contrast, they solved the problem with electrostatic field easily and efficiently that is the advantage when we introduce electrostatic field in sensor networks.

Recently, mobile sensor network node deployment based with virtual potential field is drawing researchers' more and more attentions. In [5], the authors proposed an approach, in which Delaunay triangulation was formed with these nodes, while adjacent relationship was defined if two nodes were connected in the Delaunay diagram. Force could only be exerted from those adjacent nodes within the communication range. Simulation results showed that the proposed approach had higher coverage rate and shorter convergence time than traditional virtual force algorithm. In [6], when Li and his companions were trying to overcome the connectivity maintenance and node stacking problems with traditional virtual force algorithm (VFA), they developed an extended virtual force-based approach to achieve the ideal deployment. Simulation results showed that the virtual force approach could effectively reach ideal deployment in mobile sensor networks with different ratio of communication range to sensing range. Furthermore, it achieved better performance in coverage rate, distance uniformity, and connectivity uniformity than previous VFA. In [7], they presented an energy-efficient self-deployment scheme to utilize the attractive force generated from the centroid of a sensor's local Voronoi polygon, as well as the repulsive force frequently used in self-deployment schemes with potential field. The simulation results showed that their scheme could achieve a higher coverage, leading to less sensor movements in shorter time than self-deployment schemes with traditional potential field.

In this paper, we present an adaptive deployment strategy that guarantees good coverage and uniformity, with only part of the deployment environment being given. Provided the approximate size of the deployment region, the algorithm can compute the number of nodes needed by setting appropriate parameters, thereby completing the deployment. The subsequent contents of this paper are arranged as follows: related works are introduced in Section 2.  Section 3 briefly introduces knowledge related to fluid model.  Section 4 is designed to establish mobile sensor network deployment model based on viscous fluid. Then, the model is to be solved to establish deployment algorithm.  Section 5 takes advantage of computer software to simulate the deployment algorithm, as well as its performance. Finally, Section 6 summarizes the whole paper.

## 2. Related Work

There have been plenty of representative literatures [8–12] related to wireless sensor network deployment algorithm. Sergiou and Vassiliou [8] employed a macroscopic fluid dynamic model to estimate the maximum volume of traffic that may be carried out from the sources to the sink(s) of a WSN. Gribaudo et al. [9] claimed that the behavior of large-scale WSNs was complex and difficult to analyze. Thus, they developed an analytical model of the behavior of WSNs, based on a fluid approach. Actually, they represented WSNs by a continuous fluid entity distributed in the network area. Another work [10] proposed two gas models, one of which used a virtual force approach and the other adopted akinetic approach. Pac et al. [11, 12] built hydrodynamic model for mobile sensor network, taking the entire network as fluid and mobile nodes as microelements in fluid. As for this, the problem of node deployment is transformed into a problem of hydrodynamic governing equation. Relying on the self-diffusion property of fluid, nodes, being regarded as fluid microelements, may be diffused to the deployment area along with fluid, realizing automatic deployment. The author defined the process as “self-deployment.”

The aforementioned literatures have made contributions to the deployment of mobile sensor network. Yet, they still need to be further strengthened, especially in allusion to large-scale sensor network. The deployment approach proposed in this paper is simple and pragmatic, leading to good adaptability in water, on the ground, and in the air. Literature [11, 12] provides our research with some ideologies. Based on previous researches, the paper puts forward a sensor network deployment scheme based on viscous fluid model.

Deployment algorithms mentioned in the above literatures are all based on virtual potential field. They share something in common that the deployment of mobile sensor nodes are considered as a coverage process. Nodes are affected by virtual force and eventually reach equilibrium from the initial position (randomly or prematurely set), thereby finishing the deployment of the entire network. Although these algorithms assume the deployment environment is unknown, claim themselves adaptive, and can complete the task before knowing the environment, they all implicitly presume that the size of deployment region is known, so is the number of nodes that need to be deployed. However, if the size of the deployment environment is not known a priori, these algorithms can only provide coverage to the size extent of the area that is previously fixed by the number of nodes to be deployed. Thus, a certain quality of service could not be guaranteed with these approaches. Therefore, the adaptivity and scalability of the deployment algorithms are dramatically limited.

## 3. Fluid Model

Hydrodynamics is a branch discipline of mechanics, which takes fluid as the study object, so as to research the motion of fluid, as well as the relationship and rule between acting forces. The principle of hydrodynamics may be applied in sensor networks, building a wireless sensor network based on flow field theory.

### 3.1. Viscous Fluid Motion Differential Equation

For continuous fluid, there are mainly four types of fluid models [13], where finite control volume model is divided into two types. Similarly, infinitesimal fluid micelle model is also comprised of two types. In this paper, nodes are regarded as fluid micelles that flow with fluid.

**Figure 1:**
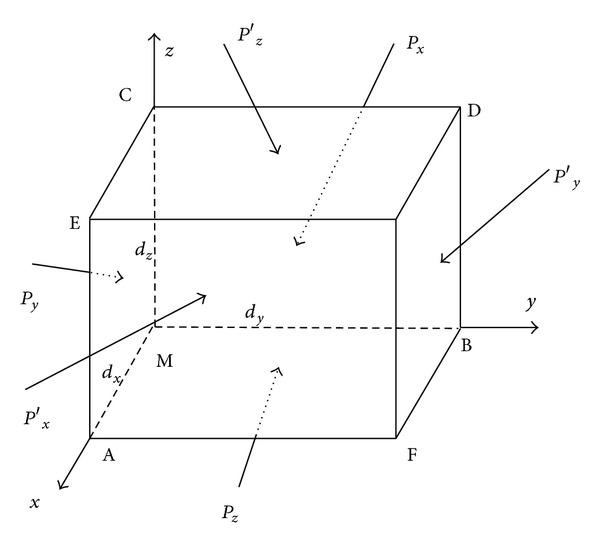
Fluid micelle force analysis.

Randomly selecting an infinitesimal fluid micelle, and assuming that its volume microelement is *dV*, fluid micelle moves along filament line, while its velocity *V* is equal to the flow velocity along the filament line. Randomly selecting a spatial point *M*(*x*, *y*, *z*) from the flow field, with the side length separately denoted by *dx*, *dy*, and *dz*, is shown in Figure 1. Assume that the velocity at point *M* at the moment is *v*(*x*, *y*, *z*, *t*), while vectors of velocity *v* along the three coordinate directions are separately *v*_*x*_(*x*, *y*, *z*, *t*), *v*_*y*_(*x*, *y*, *z*, *t*), and *v*_*z*_(*x*, *y*, *z*, *t*), and fluid density is *ρ*(*x*, *y*, *z*, *t*). In accordance with momentum conservation law, the accelerated velocity shall be *a* = *dv*/*dt*. Along the three coordinate directions, the accelerated velocities are separated and described as *a*_*x*_ = *dv*_*x*_/*dt*, *a*_*y*_ = *dv*_*y*_/*dt*, and *a*_*z*_ = *dv*_*z*_/*dt*.

External forces acting on the fluid micelle include mass force *ρ*, *f*, *d*_*x*_, *d*_*y*_, and *d*_*z*_, as well as surface forces acting on the six surfaces. There is viscous effect between micelle and fluid around, so that the surface force is normally no longer perpendicular to their respective acting surface. *P*_*x*_, *P*_*y*_, and *P*_*z*_ are employed to separately describe surface forces on the three surfaces of MBDC, MCEA, and MAFB, so that *P*_*x*_ = *p*_*x*_ *d*_*y*_ *d*_*z*_, *P*_*y*_ = *p*_*y*_ *d*_*y*_ *d*_*x*_, and *P*_*z*_ = *p*_*x*_ *d*_*x*_ *d*_*y*_. Decompose these surface forces as normal stress and shear stress, so that

(1) $$\begin{array}{c} P_{x}=p_{xx}i+p_{xy}j+p_{xz}k,\\ P_{y}=p_{yx}i+p_{yy}j+p_{yz}k,\\ P_{z}=p_{zx}i+p_{zy}j+p_{zz}k. \end{array}$$

In the equation, *p*_*xx*_, *p*_*yz*_,… separately stands for stress components, while the first subscript refers to the normal direction of acting surface and the second subscript refers to the acting direction of stress. As for this, *p*_*xx*_, *p*_*yy*_, and *p*_*zz*_ separately represent normal stress components, while the rest *p*_*xy*_, *p*_*xz*_,… are shear stress components.

Surface forces acting on the micelle also include *P*_*x*_′ = *p*_*x*_′*d*_*y*_ *d*_*z*_, *P*_*y*_′ = *p*_*y*_′*d*_*z*_ *d*_*x*_, and *P*_*z*_′ = *p*_*z*_′*d*_*x*_ *d*_*y*_, on the three surfaces of GEAF, GFBD, and GDCE. These surface forces may be decomposed into their respective normal and shear components. Compared with the aforementioned three surfaces, the three acting surfaces are slightly changed in aspect of coordinate, that is, *d*_*x*_, *d*_*y*_, and *d*_*z*_. Being expanded according to Taylor of function, and abandoning high-order small quantity, normal stress and shear stress on acting surface may be described as

(2) px′=(pxx+∂pxx∂xdx)i+(pxy+∂pxy∂xdx)j +(pxz+∂pxz∂xdx)k,py′=(pyx+∂pyx∂ydy)i+(pyy+∂pyy∂ydy)j +(pyz+∂pyz∂ydy)k,pz′=(pzx+∂pzx∂zdz)i+(pzy+∂pzy∂zdz)j +(pzz+∂pzz∂zdz)k.

According to Newton's second law, motion equation of micelle along the three coordinate directions may be figured out, while the motion equation along axis *x* shall be

(3) ρdxdydzdvxdt=fxρdxdydz−pxxdydz +(pxx+∂pxx∂xdx)dydz−pyzdzdx +(pyx+∂pyx∂ydy)dzdx−pzxdxdy +(pzx+∂pzx∂zdz)dxdy.

The above equation may be simplified as

(4) $$\begin{array}{c} \rho \frac{dv_{x}}{dt}=\rho f_{x}+\frac{\partial p_{xx}}{\partial x}+\frac{\partial p_{yx}}{\partial y}+\frac{\partial p_{zx}}{\partial z}. \end{array}$$

Put the stress component in the above equation with generalized Newton's laws of internal friction, and simplify the expression:

(5) ρdvxdt=ρfx−∂∂x[−p−23μ(∂vx∂x+∂vy∂y+∂vz∂z)+2μ∂vx∂x] +∂∂y[μ(∂vy∂x+∂vx∂y)]+∂∂z[μ(∂vx∂z+∂vz∂z)].

By expanding the second term on the right side of the equal sign, the following expression is figured out upon simplification:

(6) ρdvxdt=ρfx−∂p∂x+μ(∂2vx∂x2+∂2vy∂y2+∂2vz∂z2)=ρfx−∂p∂x+μ∇2vx.

Expression (6) is viscous fluid momentum equation, that is, Navier-Stokes equation, an important differential equation researching practical fluid.

## 4. Sensor Network Deployment Based on Viscous Fluid Model

### 4.1. Viscous Fluid Model

The property of fluid resisting the relative motion between micelles is referred to as viscidity. Assuming that there is viscidity between nodes, such viscidity is utilized to control motion between nodes, so as to maintain the connectivity of network.

Navier-Stokes equation based on viscous flow is shown in

(7) $$\begin{array}{c} \frac{dU}{dt}=f-\frac{1}{\rho }\nabla p+\nu \nabla^{2}U, \end{array}$$

where *du*/*dt* stands for velocity derivative, that is, accelerated velocity; *f* refers to vector of unit acting force, *ρ* is fluid density, and *p* represents fluid pressure, while *U* stands for the velocity of an infinitesimal fluid element. In the end, *v* is motion viscosity of fluid, and *v* = *μ*/*ρ*.

In this paper, we are only to study the motion of two-dimensional fluid. As for this, only two-dimensional vector form of Navier-Stokes equation is adopted. In rectangular coordinate system, related components may be described as

(8) $$\begin{array}{c} \frac{du}{dt}=f_{x}-\frac{1}{\rho }\frac{\partial p}{\partial x}+\nu \left(\frac{\partial^{2}u}{\partial x^{2}}+\frac{\partial^{2}u}{\partial y^{2}}\right),\\ \frac{dv}{dt}=f_{y}-\frac{1}{\rho }\frac{\partial p}{\partial y}+\nu \left(\frac{\partial^{2}v}{\partial x^{2}}+\frac{\partial^{2}v}{\partial y^{2}}\right), \end{array}$$

where *u* and *v* separately refer to velocity components of an infinitesimal fluid element along the respective direction, while *f*_*x*_ and *f*_*y*_ are component forces per unit along *x* and *y* direction. Consider

(9) $$\begin{array}{c} \frac{du}{dt}=\frac{\partial u_{i}^{t}}{\partial t}+u_{i}^{t}\frac{\partial u_{i}^{t}}{\partial x}+v_{i}^{t}\frac{\partial u_{i}^{t}}{\partial y},\\ \frac{dv}{dt}=\frac{\partial v_{i}^{t}}{\partial t}+u_{i}^{t}\frac{\partial v_{i}^{t}}{\partial x}+v_{i}^{t}\frac{\partial v_{i}^{t}}{\partial y}. \end{array}$$

Putting (9) in (8), differential equation may be figured out as

(10) $$\begin{array}{c} \frac{\partial u_{i}^{t}}{\partial t}=\frac{du}{dt}-\left(u_{i}^{t}\frac{\partial u_{i}^{t}}{\partial x}+v_{i}^{t}\frac{\partial u_{i}^{t}}{\partial y}\right),\\ \frac{\partial v_{i}^{t}}{\partial t}=\frac{dv}{dt}-\left(u_{i}^{t}\frac{\partial v_{i}^{t}}{\partial x}+v_{i}^{t}\frac{\partial v_{i}^{t}}{\partial y}\right). \end{array}$$

In (10), subscript *i* and superscript *t* are applied to identify the values of parameters *u*, *v*, *ρ*, *p*, *f*, and *ν* of fluid element *i* at the moment of *t*.

### 4.2. Analogy Relationship between Flow Field and Sensor Network

When fluid model is applied to analyze the deployment process of wireless sensor network, nodes shall be assumed with some basic properties: nodes are able to move freely; each network node is able to acquire its position, velocity, and pressure; each sensor node has a sensing range with radius of *Rs* to sense the position of obstruction, and so forth. Each sensor node has a communication radius with distance of *R*.

In addition, it is also assumed that there is viscosity between nodes, while the viscosity is related to the distance between neighbor nodes. Larger distance leads to higher viscosity, and smaller distance leads to lower viscosity. Thus, in deployment process, overdispersion of nodes may be avoided, so as to ensure the connectivity of network.

Counter definitions of the above fluid variables in mobile sensor network may be described as follows.

(*1) Velocity Vector*. Velocity vector of nodes is similar to velocity vector of viscous fluid element. The velocity of node *i* at the moment of *t* may be descried as *V*_*i*_^*t*^ = (*u*_*i*_^*t*^, *v*_*i*_^*t*^).

(*2) Density*. Local density of the position, where each node is located, may be described by *ρ*_*i*_. The definition is shown by

(11) $$\begin{array}{c} \rho_{i}=1+\frac{Rc^{2}}{{{\overset{-}{r}}_{ij}}^{2}}\times n_{i}=1+\frac{Rc^{2}n_{i}^{2}}{{\left(\sum_{j\in N_{i}}r_{ij}\right)}^{2}}. \end{array}$$

*Rc* is communication radius of node *i*, while *N*_*i*_ refers to the set of a series of neighbor nodes *j* located within this range. *n*_*i*_ refers to the number of neighbor nodes within the communication range, and *r*_*ij*_ stands for the Euclidean distance from node *i* to neighbor nodes *j* within the communication range.

(*3) Intensity of Pressure*. Intensity of pressure is the main driving entity in flowing of fluid. For ideal gas, the status equation is shown by

(12) $$\begin{array}{c} p=\rho RT, \end{array}$$

where *ρ* refers to local density, *R* is specified constant, and *T* represents absolute temperature.

(*4) Spatial Derivative of Parameter*. In (10), there are derivatives of velocity and pressure intensity, in allusion to spatial dimensionality of *x* and *y*. Grid-less method [14] is adopted to define first-order difference equation of flow variable, while the variable is identified by *ξ*. Consider

(13) $$\begin{array}{c} \frac{\partial \xi_{i}^{t}}{\partial x}=\frac{1}{n_{i}^{t}}\sum_{j\in N_{i}^{t}}\frac{\left(\xi_{j}^{t}-\xi_{i}^{t}\right)}{r_{ij}}\cos\theta_{ij},\\ \frac{\partial \xi_{i}^{t}}{\partial y}=\frac{1}{n_{i}^{t}}\sum_{j\in N_{i}^{t}}\frac{\left(\xi_{j}^{t}-\xi_{i}^{t}\right)}{r_{ij}}\sin\theta_{ij}. \end{array}$$

In (13), *θ*_*ij*_ refers to the polar angle if node *j* is in allusion to node *i*, while cos⁡⁡*θ*_*ij*_/*r*_*ij*_ and sin⁡*θ*_*ij*_/*r*_*ij*_ are weight function, where *θ*_*ij*_ = arctan⁡(*y*/*x*), $r_{ij}=\sqrt{x^{2}+y^{2}}$.

(*5) Accelerated Velocity*. In sensor networks, nodes are moving all the time. Yet, as in fluid models, the movement is not in constant velocity, which may also present accelerated velocity that is similar to fluid elements, that is, *du*/*dt*, *dv*/*dt*.

(*6) Viscosity*. Assuming that viscosity is variable, such viscosity is related to the average distance between a certain node and its neighbor nodes. Larger average distance leads to higher viscosity, while smaller distance leads to lower viscosity. As for this, viscosity may be defined as follows:

(14) $$\begin{array}{c} \mu_{i}^{t}=\frac{{\overset{-}{r}}_{ij}}{R_{c}}. \end{array}$$

In the equation, *μ*_*i*_^*t*^ refers to the viscosity of node *i* at the moment of *t*; ${\overset{-}{r}}_{ij}$ is the average distance between the node and its neighbor nodes.

### 4.3. Solving of Equation

Velocity component may be briefly and approximately transformed into

(15) $$\begin{array}{c} u_{i}^{t+\Delta t}=u_{i}^{t}+\left(\frac{\partial u_{i}^{t}}{\partial t}\right)\Delta t. \end{array}$$

Putting (10) into (15), after a minor time interval Δ*t*, the value of velocity component may be described as follows:

(16) $$\begin{array}{c} u_{i}^{t+\Delta t}=u_{i}^{t}+\left(\frac{du}{dt}-\left(u_{i}^{t}\frac{\partial u_{i}^{t}}{\partial x}+v_{i}^{t}\frac{\partial u_{i}^{t}}{\partial y}\right)\right)\Delta t,\\ v_{i}^{t+\Delta t}=v_{i}^{t}+\left(\frac{dv}{dt}-\left(u_{i}^{t}\frac{\partial v_{i}^{t}}{\partial x}+v_{i}^{t}\frac{\partial v_{i}^{t}}{\partial y}\right)\right)\Delta t. \end{array}$$

In the formula, *du*/*dt* and *dv*/*dt* may be solved with the method of smoothed particle hydrodynamics (SPH) [15]. By converting the gradient term on the right side of equal sign in Navier-Stokes momentum equation with SPH approximation method, we may be able to get the following equation:

(17) dviαdt=fα−∑j=1Nmjpi+pjρiρj∂Wij∂xiα +∑j=1Nmjμiεiαβ+μjεjαβρiρj∂Wij∂xiα.

In (17), *μ* refers to the viscosity, *ε* is shear stress rate in fluid, *f* stands for acting force, *ρ* is the density, *p* represents pressure intensity, the Greek letter superscripts *α* and *β* are designed to identify coordinate direction, and *W* refers to smoothness index, as is shown in the following equation:

(18) $$\begin{array}{c} W\left(R,h\right)=\frac{2}{\pi h^{2}}\left(\frac{3}{16}R^{2}-\frac{3}{4}R+\frac{3}{4}\right)\;\left(0\leq R\leq 2\right), \end{array}$$

where *R* = *r*/*h* = |*x* − *x*′|/*h*, *r* is the distance between two points or particles on *x* and *x*′, and *h* refers to the smooth distance, that is, communication radius. Moreover, *ε* approximate expression of particle *i* shall be

(19) εiαβ=∑j=1Nmjρjvjβ∂Wij∂xiα+∑j=1Nmjρjvjα∂Wij∂xiα −(23∑j=1Nmjρjvj·∇iWij)δαβ.

As for this, *du*/*dt*, *dv*/*dt* may be worked out. In the computation, *du*/*dt*, *dv*/*dt*, *V*_*i*_^*t*^, *ρ*_*i*_, *p*, ∂*ξ*_*i*_^*t*^/∂*x*, ∂*ξ*_*i*_^*t*^/∂*y* may be put into (19) accordingly. As for this, the value of velocity component may be figured out by iterative computation.

### 4.4. Constraint Condition, Initial Condition, and Physical Boundary Condition

**Figure 2:**
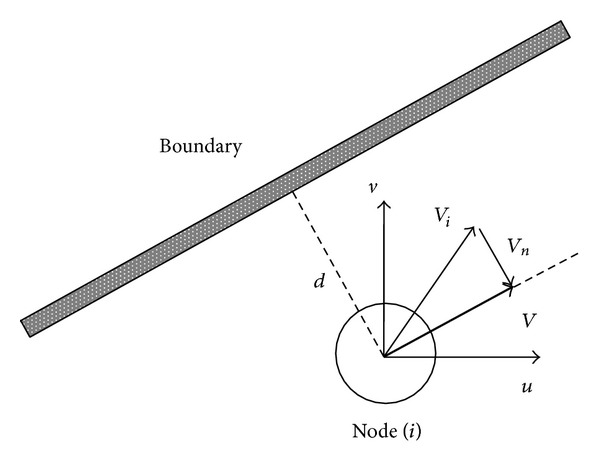
Object surface boundary condition.

(*1) Initial Value*. Time iteration method in (16) needs to acquire the initial value of velocity component in advance, as well as current position before calculating the position of the next step.

(*2) Physical Boundary Condition*. The velocity of nodes sticking closely to object surface is tangent to object surface; that is, flow on object surface is tangent to object surface. As is shown by Figure 2, if the distance from nodes to obstruction or boundary is smaller than *d*, the moving velocity of node is changed.

(*3) Constraint and Control Condition*. Denoting the threshold value of velocity as *V*_th_, the velocity of sensor node shall be controlled below the threshold.

### 4.5. Deployment Algorithm

Process of the algorithm is shown in Algorithm 1.

## 5. Simulation and Results

The section is designed to simulate the deployment algorithm under two different situations: with obstruction and without obstruction, with initial status node deployment diagram, final status node deployment diagram, coverage rate changing curve, and uniformity changing curve presented.

### 5.1. Coverage

Generally, coverage can be considered as the measured service quality of a sensor network. In research on multirobot system, Gage firstly proposed the concept of coverage degree [16]. Literature [17] defines coverage rate as the specific value between the total coverage area of all nodes and the total area of the target region, as is shown by

(20) $$\begin{array}{c} \text{Coverage}=\frac{{⋃}_{i=1,\ldots ,N}A_{i}}{A}, \end{array}$$

where *A*_*i*_ is the area covered by the *i*th node, *N* is the total number of nodes, and *A* stands for the area of the region.

### 5.2. Uniformity

Good uniformity is a perfect standard to measure the service life of a network. Literature [17] defines uniformity as the standard deviation of distance between nodes. Smaller standard deviation leads to higher uniformity of network coverage. Consider

(21) $$\begin{array}{c} U=\frac{1}{N}\overset{N}{\underset{i=1}{\sum }}U_{i},\\ U_{i}={\left(\frac{1}{K_{i}}\overset{K_{i}}{\underset{j=1}{\sum }}{\left(D_{i,j}-M_{i}\right)}^{2}\right)}^{1/2}, \end{array}$$

where *N* is the total number of nodes. *K*_*i*_ is the number of neighbors of the *i*th node, *D*_*i*,*j*_ is the distance between *i*th and *j*th nodes, and *M*_*i*_ is the mean of internodal distances between the *i*th node and its neighbors.

In the calculation of the local uniformity *U*_*i*_ at the *i*th node, only neighboring nodes that reside within its communication range are considered. Uniformity measure is a local measure and is computed locally because each node has access to local information only. A smaller value of *U* means that nodes are more uniformly distributed.

### 5.3. Results

In practical application, owing to weather factor, battery failure, or other reasons, some nodes may be neutralized. Failure nodes may reduce the coverage rate of certain regions. When this happens, the balance of node deployment algorithm based on flow field model may be damaged, urging nodes to be relocated to recover these “exposed” regions, so as to regain a new balanced state.

In this paper, 3D topographic map is applied to vividly describe the simulation process. Simulation parameters are shown in Table 1.

**Figure 3:**
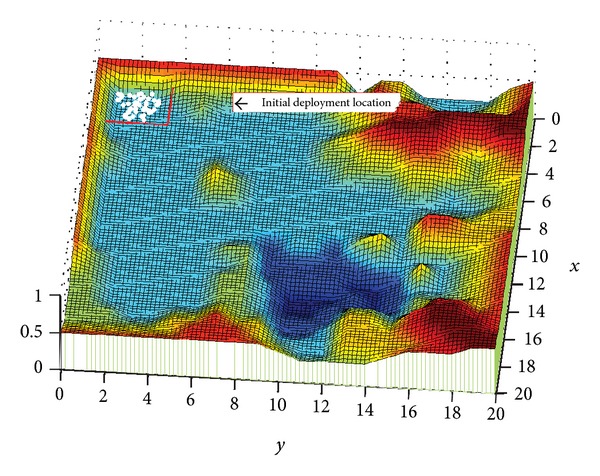
Node deployment environment and initial position.

As is shown by Figure 3, this is a 3D topographic map simulated by computer software Matlab. In the map (Region A), there are mountainous area (in red), lake (in dark blue), and plain land. The initial deployment position of nodes is a certain corner in the map, that is, the range shown by red rectangle. The white dots are mobile nodes to be deployed.

Within the sensing radius *R*_*s*_, nodes are able to detect the environment and to collect information. Similarly, they are also able to sense acting forces from neighbor nodes. The communication radius of nodes is larger than sensing radius *R*_*s*_. Nodes are enabled to freely and mutually exchange information within the communication radius. The initial position of nodes is located at the lower left corner of the map. When the deployment begins, affected by unbalanced resultant force, all nodes will move to other positions in Region A.

**Figure 4:**
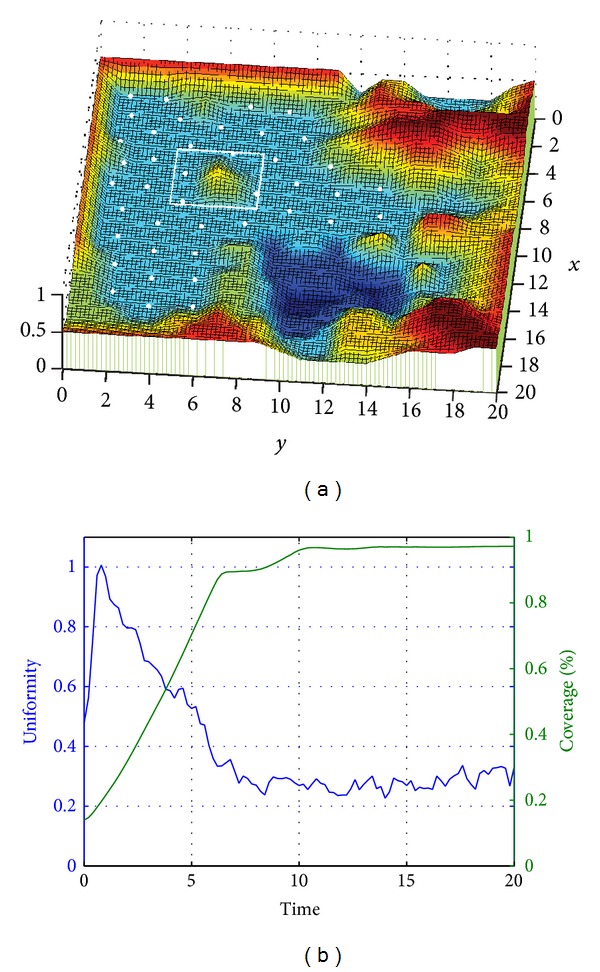
(a) Node position after deployment and (b) network coverage rate and uniformity Curve.

Figure 4 shows the deployment result based on the algorithm proposed in this paper. According to the figure, nodes are distributed in plain lands, avoiding high mountains, massifs, lakes, and other obstructions. In the figure, the white rectangle shows a massif, while nodes are located around it. Seeing from the left plot, owing to the obstruction, nodes have to evade to complete the deployment. This is quite pragmatic in practical application. Dangers or inaccessible areas are avoided to reduce unnecessary node loss and waste, demonstrating the self-adaption nature of the algorithm. The right plot shows variation of coverage and uniformity in the deployment process. It may be discovered that, owing to the existence of obstruction, nodes are unable to fully cover the whole region, so that the maximum value of coverage rate is smaller than 1.

**Figure 5:**
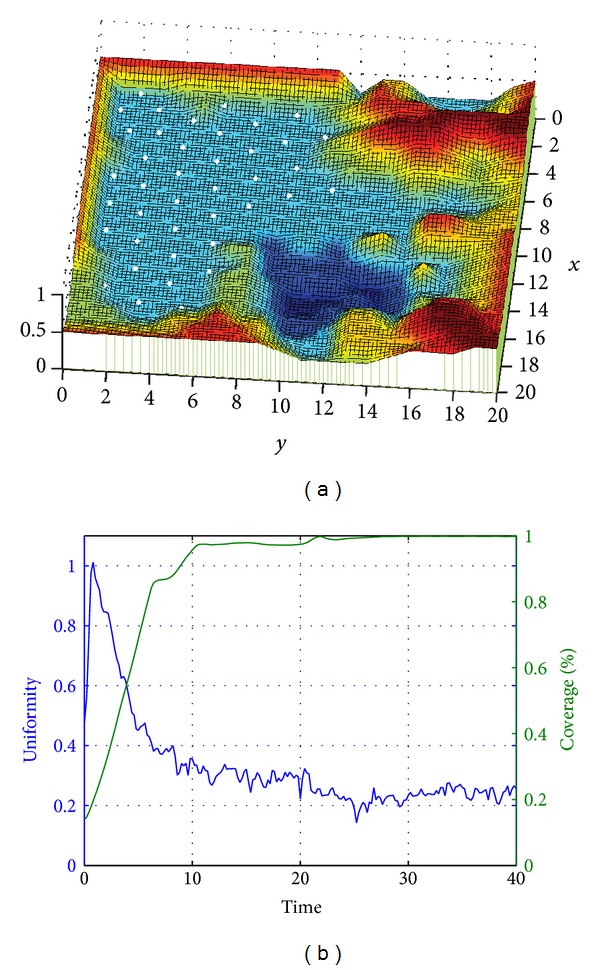
(a) Redeployment effect after the massif disappears and (b) network coverage rate and uniformity curve.

In Figure 5, massif mentioned in Figure 4 is manually removed to assess the self-adaptability of nodes. According to the figure, nodes' balance state is damaged, and nodes are relocated, reaching a new balance state. After the massif disappears, there is no coverage gas left. Nodes around successfully recovered the area.

Seeing from the figure, there are nodes moving towards the place. After the adjustment, nodes regained the balance. Referring to coverage and uniformity curve in the right plot, we may clearly understand the process. When time = 20 (pointed by the arrow in the figure), the coverage rate increases from below 1 to 1. In the meanwhile, uniformity is also sharply improved. All these consequences resulted from the disappearance of massif.

After the redeployment (20 < time < 40), nodes reach the balance state again. The value of coverage increases to approximately 1, while the value of uniformity is stabilized around 0.2, which is slightly lower than the previous stable value −0.4. Thus, the disappearance of massif improves the uniformity.

Based on the above researches on deployment algorithm, this algorithm is properly simplified based on predecessors' research findings, eliminating the complexity of previous similar algorithms while preserving the property of self-adaption.

## 6. Conclusion

Deployment of large-scale sensor network is always a research hot spot in the field. How to save time and energy, to uniformly deploy nodes, so as to maximize service life of network? This is a goal pursued by all deployment algorithms. However, in practical application, problems and difficulties encountered are complicated and diversified. As for this, an extensible, self-adaptive, robust, and simple deployment algorithm is necessary.

In this paper, viscous fluid model is applied in deployment of wireless sensor network, with an extensible sensor network deployment algorithm proposed. This algorithm abstracts sensor nodes as fluid particles, while the parties abide by the motion rules of fluid. Nodes simulate the diffusion and self-propagating behavior of particles in fluid, realizing effective and ideal coverage range, so as to complete network deployment in unknown area. Shown by the simulation test, the algorithm shows good performance in various situations. For completely unknown deployment area, the algorithm proposed in this paper may fully bring to play its superiority of self-adaption, so as to reach the expected deployment effect. Thus, it is a robust and self-adaptive deployment algorithm.

## Figures and Tables

**Algorithm 1:**
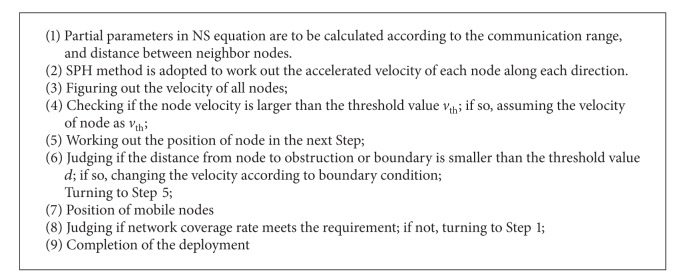
Node deployment algorithm based on flow field model.

**Table 1:** Simulation parameters.

Parameters	Values
Region A	20 m × 20 m

Sensor Numbers (*N*)	100
Communication radius (*Rc*)	2 m
Sensoring radius (*Rs*)	1 m
Distance threshold (*d*)	0.5 m
Simulation time step (Δ*t*)	0.05
Velocity threshold (*v*_th_)	2
Pressure (*p*)	3

## References

[1] Howard A, Matarić MJ, Sukhatme GS (2002). Mobile sensor network deployment using potential fields: a distributed, scalable solution to the area coverage problem. *Distributed Autonomous Robotic Systems 5*.

[2] Zou Y, Chakrabarty K Sensor deployment and target localization based on virtual forces.

[3] Shucker B, Bennett JK (2007). Scalable control of distributed robotic macrosensors. *Distributed Autonomous Robotic Systems*.

[4] Toumpis S, Tassiulas L Packetostatics: deployment of massively dense sensor networks as an electrostatics problem.

[5] Yu X, Huang W, Lan J, Qian X A novel virtual force approach for node deployment in wireless sensor network.

[6] Li J, Zhang B, Cui L, Chai S (2012). An extended virtual force-based approach to distributed self-deployment in mobile sensor networks. *International Journal of Distributed Sensor Networks*.

[7] Larrabide I, Kim M, Augsburger L, Villa-Uriol MC, Rüfenacht D, Frangi AF (2012). Fast virtual deployment of self-expandable stents: method and *in vitro* evaluation for intracranial aneurysmal stenting. *Medical Image Analysis*.

[8] Sergiou C, Vassiliou V (2013). Estimating maximum traffic volume in wireless sensor networks using fluid dynamics principles. *IEEE Communications Letters*.

[9] Gribaudo M, Chiasserini C-F, Gaeta R, Garetto M, Manini D, Sereno M A spatial fluid-based framework to analyze large-scale wireless sensor networks.

[10] Wesley K, Spears D, Spears W, Thayer D (2005). Two formal gas models for multi-agent sweeping and obstacle avoidance. *Formal Approaches to Agent-Based Systems*.

[11] Pac MR, Erkmen AM, Erkmen I Scalable self-deployment of mobile sensor networks: a fluid dynamics approach.

[12] Pac MR, Erkmen AM, Erkmen I Towards fluent sensor networks: a scalable and robust self-deployment approach.

[13] Anderson J D (1995). *Computational Fluid Dynamics*.

[14] Liu GR, Liu MB (2003). *Smoothed Particle Hydrodynamics: A Meshfree Particle Method*.

[15] Monaghan JJ (2005). Smoothed particle hydrodynamics. *Reports on Progress in Physics*.

[16] Douglas WG (1992). Command control for many-robot systems. *Unmanned Systems*.

[17] Heo N, Varshney PK A distributed self spreading algorithm for mobile wireless sensor networks.

